# Multi-level aggregation analysis of microbiome composition and host gene expression reveals associations with systemic and local immunity

**DOI:** 10.1128/spectrum.03593-25

**Published:** 2026-08-10

**Authors:** Lev I. Shagam, Anna Elizarova, Yukihide Momozawa, Julia Dmitrieva, Rob Mariman, Souad Rahmouni, Edouard Louis, Michel Georges, Alexander V. Tyakht, Natalia Klimenko

**Affiliations:** 1 Unit of Animal Genomics, GIGA Institute and Faculty of Veterinary Medicine, Avenue de l'Hôpital, University of Liège 401260, Liège, Belgium; 2 Institute of Gene Biology, Russian Academy of Sciences 164227 , Moscow, Russia; 3 Department of Gastroenterology, Faculty of Medicine, CHU & GIGA Institute, Avenue de l'Hôpital, University of Liège 401260, Liège, Belgium; 4 WELBIO Department, WEL Research Institute 630407 , Wavre, Belgium; China Agricultural University, Beijing, China

**Keywords:** gut microbiome, host gene expression, immunity, *Catenibacterium*, hierarchical feature aggregation

## Abstract

The human gut microbiome plays a critical role in immune regulation, yet the molecular links between microbiome composition and host gene expression remain incompletely understood. We analyzed associations between host gene expression and microbiome composition in a cohort of 315 healthy individuals, integrating microarray-based gene expression data from three intestinal sites (ileum, transverse colon, and rectum) and six immune cell types with microbiome sequencing data. Using a hierarchical feature aggregation strategy combining principal component analysis, clustering, and covariate correction, we discovered significant associations primarily related to immunity. While microbial profiles were similar across the three intestinal sites, the transverse colon yielded the most “microbiome-host gene expression” associations. Among the immune cell types, CD8+ cells showed the highest number of associations. The first principal component of microbiome composition, reflecting a gradient from commensals (e.g., *Ruminococcaceae* and *Christensenellaceae*) to proinflammatory taxa ([*Ruminococcus*] *gnavus* and *Lachnoclostridium*), correlated with the expression of TNF-α-linked genes (*HMOX1*, *CPI17*, *HSD3B2*, and *SLC5A1*). Among individual genera, *Catenibacterium* abundance was associated with gene expression in both intestinal and immune cells, including negative associations with MRPS21 (related to mitochondrial function) in the transverse colon and with CD8+ gene programs related to T cell differentiation. These findings align with emerging evidence implicating mitochondrial dysfunction in intestinal inflammation. Our results identify multi-level associations between the gut microbiome and host gene expression, suggesting potential mechanisms by which microbiota shape local and systemic immunity and vice versa. The implicated genes and taxa represent candidates for experimental validation to improve understanding of host-microbiome homeostasis and its disruption in disease.

**IMPORTANCE:**

The gut microbiome and immune system are engaged in a complex interplay throughout human life. While most associative studies focus on case-control comparisons—typically examining patients with conditions such as inflammatory bowel disease or metabolic diseases—less is known about the molecular links between the microbiome and immune system in healthy individuals. In this study of a large cohort of healthy individuals, we addressed this gap by applying multiscale modeling to tackle the high dimensionality of host-microbiome data. We identified multi-level associations between microbiome composition and host gene expression in both intestinal tissues and immune cells. These findings offer a valuable reference for understanding baseline host-microbiome communication and highlight molecular candidates—such as TNF-α-related genes and mitochondrial pathways—for future experimental validation.

## INTRODUCTION

The gut microbiome plays a pivotal role in the functioning of the host organism. Its composition is associated with the host metabolome, as well as with risks, occurrence, and severity of various diseases. One particularly intriguing aspect is the relationship between the gut microbiome and the host’s immune system. According to recent studies, these interactions influence not only the local structures of the immune system but also systemic immune response (1–4). Interestingly, despite being separated from myriads of live bacteria just by a single-cell-thick intestinal wall, a healthy human organism maintains a “tolerance-protection” balance via the fine-tuning of the immune system. How exactly the immune system manages this, despite the observed immense variability of microbiome compositions and absence of a reference “healthy” community, is still being clarified (5). Disruptions of this delicate equilibrium can contribute to the onset of severe pathologies, such as inflammatory bowel diseases (IBDs).

One direct approach to quantifying interactions at the “host-microbiome” interface and evaluating the local immune mechanisms is to compare host gene expression with microbiome composition. In mice, microbiota was shown to induce transcriptional response in intestinal epithelial cells affecting around 10% of the host’s genes (6). Many human studies have explored similar interactions in various diseases marked by immune system dysfunction—and most of the identified associations were related to immunity. A longitudinal study of IBS patients (7) integrated multi-omics data, revealing a network of correlations among “host genes, microbial taxa, and metabolites”. The two major forms of IBD—ulcerative colitis and Crohn’s disease (CD)—are known to manifest pronounced microbiome alterations and, besides clinical differentiation, can be distinguished by the microbiome profiles with an accuracy far better than chance. However, the colon mucosa transcriptomes were surprisingly similar between the two diseases (8). In a large multi-omics survey of IBD patients and their microbiome (9), it was observed that host gene expression in the gut was primarily determined by intestinal site (ileum vs rectum) rather than disease phenotype and inflammation. Associations between microbiome composition and host gene expression were site-specific, with several commensal taxa linked to human genes encoding antimicrobial proteins, including DUOX2, which is upregulated in IBD. Interestingly, DUOX2 expression was also associated with microbiome composition in another illustrative example of a disease with severely disrupted barrier function—cystic fibrosis (10). Another study investigating the disruption of host-microbiome interactions in IBD revealed tissue-specific links between intestinal microbes and inflammation-related genes. The findings included a correlation between the *Erysipelotrichaceae* family and collagen biosynthesis pathways, as well as a correlation between anaerobic butyrate-producing taxa and genes involved in transmembrane transport, host interleukin signaling, and metal ion response (11).

Furthermore, to increase the understanding of how gut microbiome affects systemic host homeostasis, it is important to examine the connections between microbiome composition and immune status parameters. In a longitudinal study of cancer patients undergoing hematopoietic cell transplantation, consistent correlations were found between the dynamics of immune cell counts and specific microbial genera in the gut (12). However, a deeper investigation of the immune transcriptome in humans has yet to be undertaken.

As highlighted by the disorder-focused studies mentioned above, the direction of host-microbiome links was clearly dictated by the nature of the condition. It is worth mentioning that these connections may have been confounded by secondary factors such as prior medical treatments, thus obfuscating the ability to identify the meaningful associations. The issue is further complicated by the high dimensionality of both host expression and microbiome community features, which, via the multiple comparison correction, reduces the sensitivity of discovery.

Here, we addressed this gap by comparing mucosal microbiome communities with matching host gene expression profiles at distinct sites along the intestinal tract (ileum, transverse colon, and rectum), as well as in multiple immune cell types within a large cohort of healthy subjects (*N* = 315). Utilizing a multi-level hierarchical feature aggregation approach to manage the high-dimensionality challenges, we discovered significant associations related to immunity and host metabolism that can shed light on local and systemic interactions between humans and their commensal microbes.

## RESULTS

### Establishing robust microbiome profiles based on multiple gut sites and 16S rRNA regions

Although stool samples are commonly used for human microbiome surveys due to their ease of collection, they provide a mixed signal, reflecting the luminal community of the distal colon. For a more accurate investigation of potential links between microbiome composition and human gene expression in the gut, we utilized biopsy samples from three distinct intestinal sites—the ileum, transverse colon, and rectum (previously published data [13]). Furthermore, since 16S rRNA amplicon sequencing is known to produce different profiles depending on the amplified variable region (14), we analyzed each sample using three regions (see Materials and Methods).

To assess the variability of observed microbiome composition across these target gene regions, we compared the abundance of microbial genera between the different amplicons in a pairwise manner. First, the amplicons varied by the presence of potential contaminants (contaminant detection strategy is described in reference 13, Table S1): the V34 region was less prone to contamination than V12 (*P* = 2.76 × 10^−17^, Mann-Whitney test) and V56 (*P* = 1.85 × 10^−11^), while no significant difference was observed between the latter two (*P* = 0.17) (Fig. S1B). However, the V34 region contained the highest proportion of reads mapping to human DNA (Fig. S1A). The observed variability in microbiome composition across amplicons was primarily due to the low-abundance genera (Fig. S1C). After filtering them out (see Materials and Methods), the median Spearman correlation between amplicons ranged from 0.73 to 0.78. Comparison of taxon abundance across amplicons showed borderline significance when comparing V1V2–V5V6 against V3V4–V5V6 (Mann-Whitney *U* test for Pearson correlation coefficients, *P* = 0.058), while no significant differences were observed for the other amplicon pair combinations (Fig. S1D). After applying centered-log ratio (clr) transformation, correcting for phenotypic and batch factors and filtering, the overall variability of microbiome composition explained by the intestinal site was relatively low (3.3% of the total variance, Fig. S2). Additionally, linear regression revealed that no feature was significantly associated with the intestinal site (*P* > 0.05). Consequently, we performed averaging across all intestinal sites and amplicons after correction using linear regression (see Materials and Methods). This refined microbiome composition profile for each subject was then used for the association analysis with the host gene expression.

### Microbiome composition at different levels of hierarchical feature aggregation

**Fig 1:**
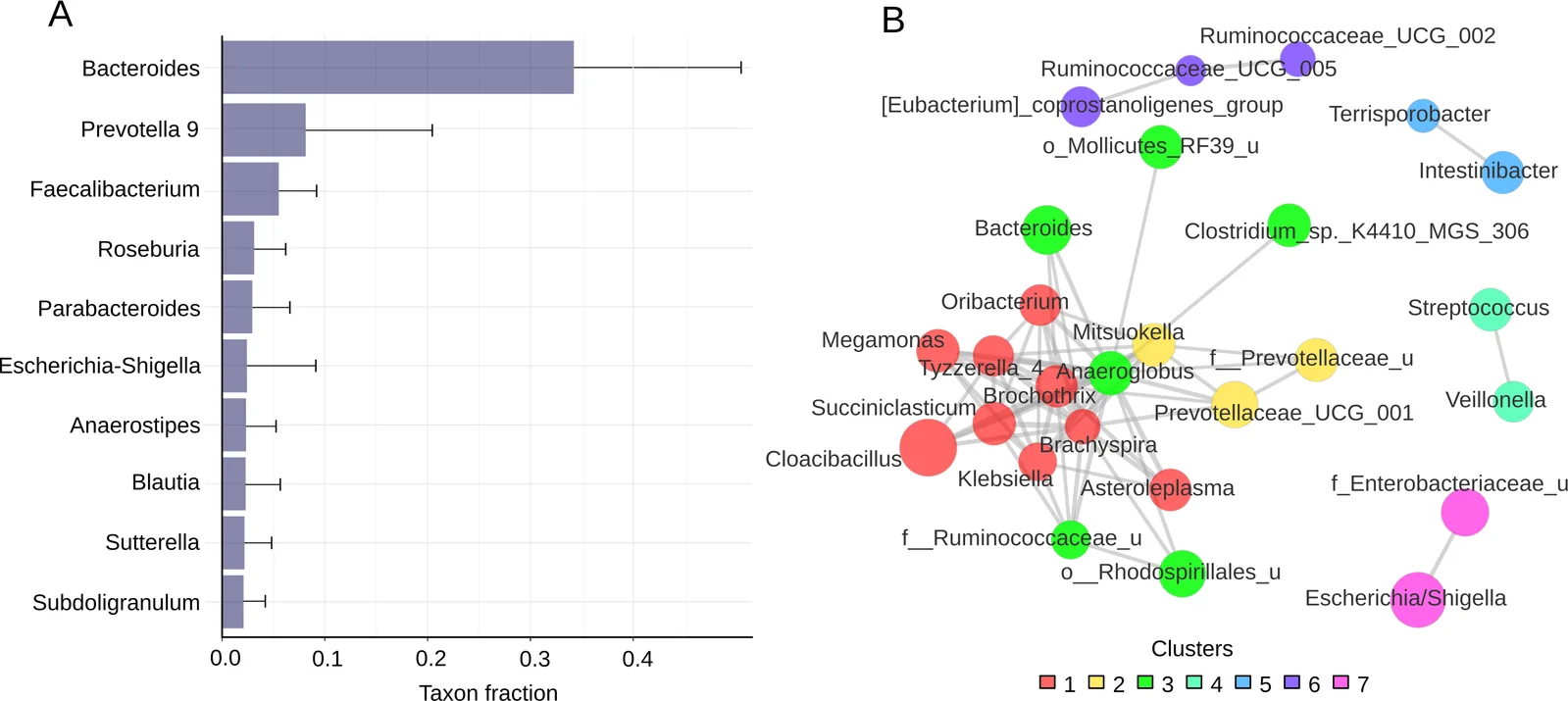
Microbiome composition of biopsy samples at the genus and microbial cluster levels. (**A**) Relative abundances of the top 10 genera averaged across intestinal sites. (**B**) Clusters identified by clustering the genera using the Louvain algorithm (see Materials and Methods). Vertices represent genera, with edges connecting pairs of highly correlated genera. Vertex colors indicate cluster membership; vertex size is proportional to the mean abundance of the corresponding genus. “_u” denotes unclassified genera from a higher taxonomic clade. Singletons are not shown.

The microbiome composition of the microbiome was dominated by the genera *Bacteroides* (34.2%), *Prevotella* (8.1%), and *Faecalibacterium* (5.5%) (Fig. 1A). These genera are typical members of the healthy human gut microbiome, as detected in both biopsy and stool sample studies (15–17).

Aggregation of samples by applying enterotyping methodology revealed four enterotypes: three driven by *Bacteroides* and one by *Prevotella* (Fig. S3). The average abundances of genera in enterotypes were consistent with those observed in stool samples from the TwinsUK and MetaCardis cohorts (18, 19). There were two enterotypes with strongly dominating *Bacteroides* (>40%), distinct by their evenness of major taxon abundance; we denoted them as Bact1 (higher evenness) and Bact2 (lower evenness) in accordance with reference 19. The third *Bacteroides*-dominated enterotype had lower *Bacteroides* abundance compared to Bact1 and Bact2, alongside higher *Ruminococcaceae* abundance, resembling the Rum enterotype in reference 19. The enterotype dominated by *Prevotella* was labeled Prev.

We further clustered co-abundant genera into microbial clusters (“cooperatives”) (20), reducing 145 genera into 7 clusters and 118 singletons (Fig. 1B). The correlation structure of the data partially mirrored previously published correlation graphs based on stool sample microbiome data. Particularly, we observed the described co-occurrence of *Terrisporobacter* and *Intestinibacter*, [*Eubacterium*], and *Ruminococcaceae* UCGs, as well as *Veillonella* and *Streptococcus* (20–22).

Principal component analysis (PCA) of the microbiome composition allowed us to extract 15 leading microbiome principal components (mPCs), collectively explaining 44.1% of the composition variation. To minimize the inter-individual variability, it is expedient to consider microbiome composition after correcting for its main principal components. This approach, actively applied in genetics (23–25), could help adjust for the multimodality of human gut microbiome data—thus aiding in uncovering more subtle effects. Following this approach, we adjusted the genus abundances using the first 15 mPCs through a linear model and referred to these adjusted features as mPC-corrected genus abundances.

In summary, we derived four types of microbiome composition features: (i) enterotypes, (ii) mPCs, (iii) abundances of clusters and singleton genera, and (iv) mPC-corrected genus abundances.

### Gene expression data on different levels of feature aggregation

As a measure of host gene expression in the same subjects, we evaluated transcriptome microarray data from tissue biopsies at three intestinal sites and six types of immune cells, as previously published (25). In addition to the originally published gene expression principal components (gPCs) and gene expression values corrected for several gPCs (gPC-corrected gene expression), we clustered the genes based on co-expression, yielding 15–48 clusters per intestinal site or cell type (see Materials and Methods). The resulting list of transcriptomic features included: (i) gPCs, (ii) gPC-corrected gene expression, and (iii) expression of gene clusters and singleton genes.

The most highly expressed genes varied across different intestinal sites and immune cell types (Fig. S4). In the intestinal tissues, among the top genes expressed were those involved in immune response regulation (two *HLA* genes in the rectum and ileum), maintenance of the mucosal layer (mucin genes *MUC1* in the rectum and *FCGBP* in the transverse colon), detoxification (two *UGT* genes in the transverse colon; metallothionein genes in both the transverse colon and rectum), as well as cell proliferation and differentiation (*OLFM4* and *regenerating* genes in the ileum). In the immune cells, the most highly expressed genes included those related to general processes such as transcription and translation (*RPS26*, *HNRNPH1*, *RPL8*, and *SRSF5*), as well as genes with immune-specific functions: *CD3G*, essential for T cell receptor signaling; *STAT1*, which mediates immune response to pathogen infections in T cells; and lysozyme-encoding genes important for antibacterial defense. In platelets, the top expressed genes were involved in secretion processes (*PIP4K2A* and *SRGN*) and associated with cytoskeleton formation and function (*TUBB1* and *ACTB*).

### Associations between microbiome composition and gene expression

**Fig 2:**
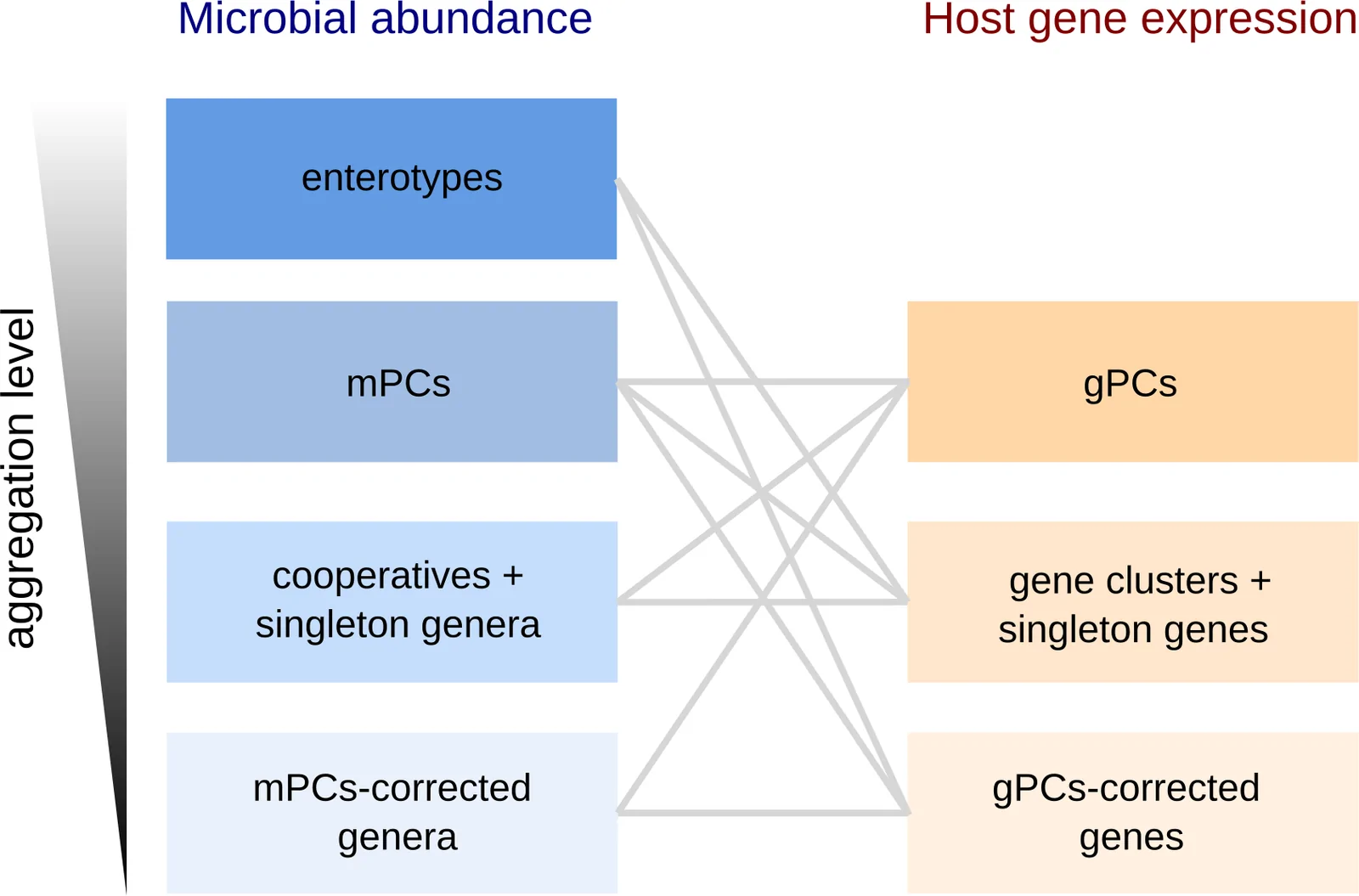
Outline of analyses conducted to identify associations between microbiome and host gene expression. Bluish boxes denote microbiome features, beige boxes denote host transcriptomic features, and gray lines denote comparisons conducted between levels of microbiome and host transcriptomic features.

Having established features at various levels of hierarchical feature aggregation (aggregation levels), we used them to investigate associations between microbiome composition and host gene expression (three features for the transcriptome and four for the microbiome, resulting in nine comparisons, as shown in Fig. 2).

**Fig 3:**
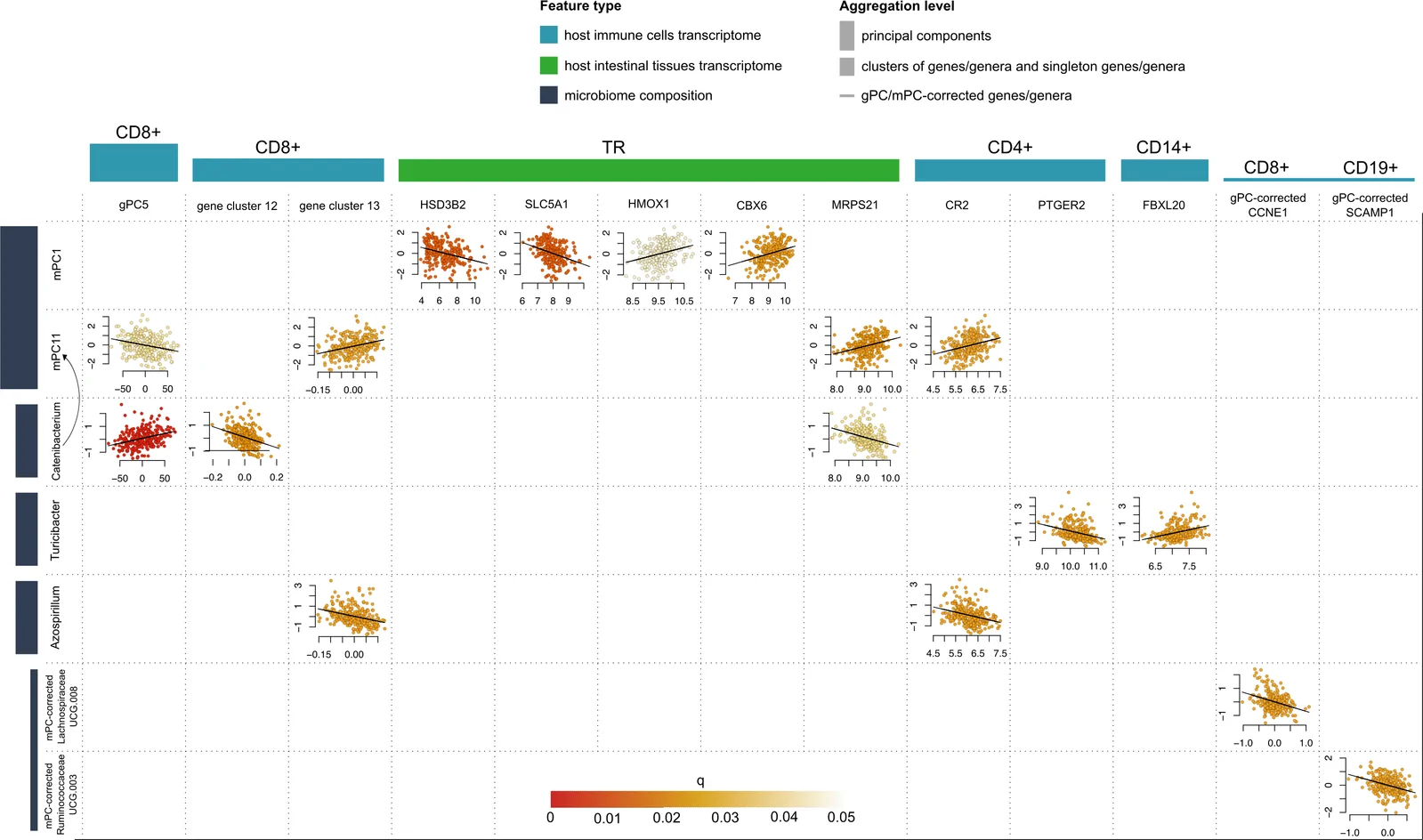
Associations between microbiome composition and host gene expression at different aggregation levels. The height of the top bars and the width of the left-side bars show the level of hierarchical feature aggregation, respectively; their colors correspond to feature type. Scatterplots show significant associations (*q* < 0.05) with the points colored by the *q* value. For compactness, among the uncorrected singleton genera, only those involved in ≥2 associations are shown (the rest three associations are provided in Fig. S5). TR, transverse colon.

We discovered 20 significant associations (*q* < 0.05); the majority of them (*N* = 17) are shown in Fig. 3, and the three remaining associations (each involving one bacterial genus) are for compactness provided in Fig. S5. Additionally, we listed these and non-significant results with 0.05 < *q* < 0.15 (*N* = 32) in Table S2 to offer insights for future studies.

### Primary microbiome axis is mainly associated with the expression of immune-related genes in the transverse colon

**Fig 4:**
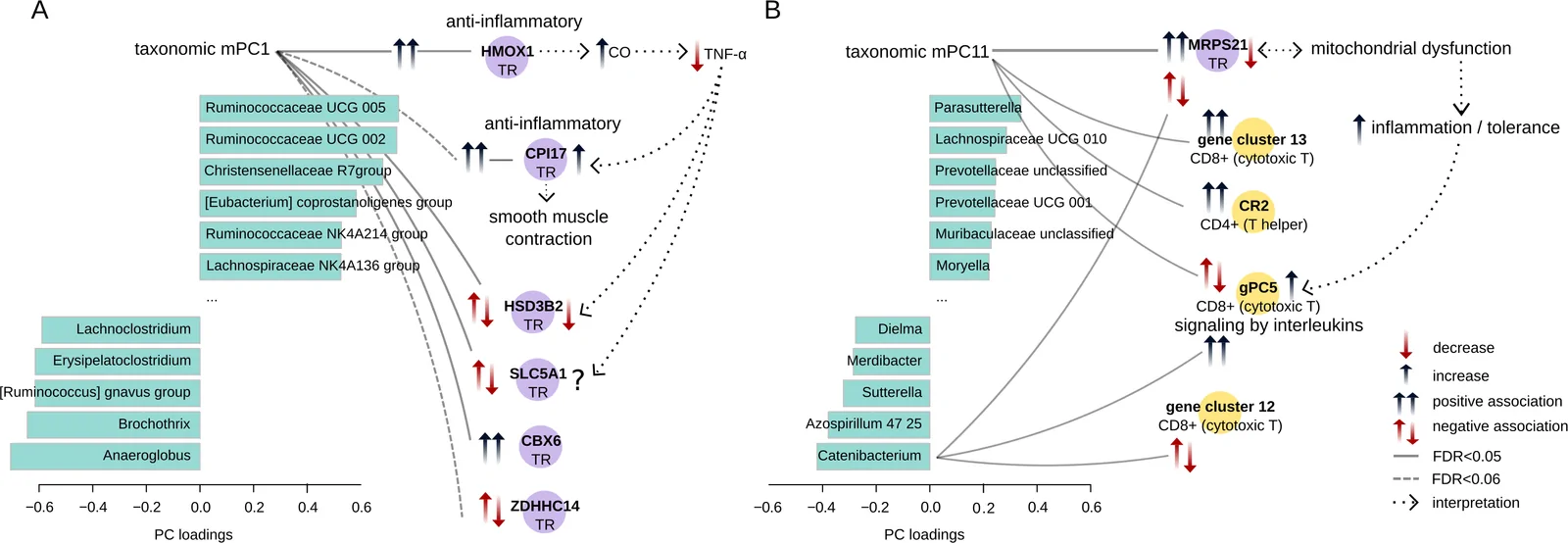
Hypothesized involvement of bacterial taxa in the regulation of immunity and inflammation, viewed through the lens of taxonomic principal components. Loadings are shown for mPC1 (**A**) and mPC11 (**B**) significantly associated with host gene expression. Circles represent genes labeled with their intestinal site or cell type (below the gene names; TR, transverse colon). Solid and dashed lines denote associations that are significant (*q* < 0.05) or on the edge of significance (0.05 ≤ *q* < 0.06), respectively. Dotted lines highlight interpretations described in the text, with arrows indicating hypothesized causal relationships. Black co-directional and red oppositely directed arrows denote positive and negative associations between an mPC and host gene expression feature, respectively. At the end of dotted arrows, single red and black arrows near metabolites/processes/genes indicate the direction of change for that feature, based on the corresponding change in the starting feature.

The analysis of associations between microbiome principal components and gene expression data yielded eight significant results (*q* < 0.05, Fig. 3, top two rows). Half of them involved gene expression in the transverse colon and the first microbiome principal component (mPC1). The mPC1 reflects a gradient of taxa from positive loadings dominated by unclassified *Christensenellaceae*, *Ruminococcaceae,* and [*Eubacterium*] *coprostanoligenes* group to negative loadings dominated by *Anaeroglobus*, *Brochothrix,* and [*Ruminococcus*] *gnavus* group (Fig. 4A) (here [*Ruminococcus*] in brackets refers to *Ruminococcus* from the *Lachnospiraceae* family later reclassified as *Mediterranibacter*). In the transverse colon, the expression of *HSD3B2* (raw *P* = 9.8 × 10^−8^, *q* = 0.0079) and *SLC5A1* (*P* = 7.1 × 10^−8^, *q* = 0.0079) was negatively associated with mPC1, while *HMOX1* (*P* = 1.3 × 10^−6^, *q* = 0.0444) and *CBX6* (*P* = 4.1 × 10^−7^, *q* = 0.0166) showed positive associations. Notably, *HSD3B2*, *SLC5A1*, and *HMOX1* are known to play roles in the intestinal immune response (see Discussion). No significant associations between mPC1 and gene expression were found in any other intestinal site or cell type. Interestingly, among top marginally significant associations for mPC1, all were also found in the transverse colon (0.05 < *q* < 0.15, *N* = 5: *OLFM4*, *SLC15A1*, *CPI17*, *ZDHHC14*, and *ZBTB11-AS1*, Fig. S6).

Despite the fact that the mPC1 reflects the main variation in microbiome data, similar to enterotypes, we found no significant differences in gene expression between the enterotypes (*q* > 0.15).

### *Catenibacterium* abundance is related to systemic immunity and local inflammation

At the level of individual and clustered genera, *Catenibacterium* showed the highest number of significant associations (*n* = 3) with host gene expression data. Notably, it was linked to both intestinal (transverse colon biopsy) and blood immune cells (CD8+). In CD8+ immune cells, the genus abundance correlated positively with the fifth gene principal component (gPC5) (*P* = 9.9 × 10^−8^, *q* = 0.0025) and negatively with gene cluster 12 (*P* = 7.9 × 10^−9^, *q* = 0.0150) (Fig. 3). gPC5 in CD8+ cells was enriched for genes involved in the “signaling by interleukins” pathway (Table S3), whereas gene cluster 12 was enriched for pathways involved in rRNA processing and translation (reactome: R-HSA-8868773, R-HSA-6791226, R-HSA-72312, R-HSA-72766, and R-HSA-6804754) (Table S4). Interindividual variation in these transcriptomic features may reflect differences in the proportions of CD8+ T cell states or variability in the activity of their differentiation (26). Although the broad nature of these features makes it difficult to infer the specific role of *Catenibacterium* in these processes, its abundance is clearly associated with systemic immune response.

Additionally, *Catenibacterium* was negatively associated with the expression of MRPS21 in the transverse colon (*P* = 2.7 × 10^−8^, *q* = 0.0371), which may indicate a downregulation of mitochondrial metabolism, which is related to intestinal inflammation (27–29). The associations observed for *Catenibacterium* aligned closely with those seen for the mPC11, as this genus serves as the primary negative loading for that principal component (Fig. 3 and 4B).

Additionally, five bacterial genera manifested one to two associations with the host features. *Turicibacter* was positively associated with *FBXL20* expression in CD14+ cells and negatively associated with PTGER2 in CD4+ cells. *Azospirillum 47_25* genus was negatively associated with *CR2* expression in CD4+ cells and with gene cluster 13 in CD8+ cells (*q* = 0.019 each). One immune cell-related association was discovered for each of the genera classified as *Clostridium innocuum* group, Clostridiales vadin BB60 group, and *Dielma* (Table S2 and Fig. S5).

### Correction for principal components uncovers novel associations

After correcting for the main principal components (mPCs and gPCs), we identified two significant associations (Fig. 3). The mPC-corrected *Lachnospiraceae UCG-008* abundance was negatively associated with gPC-corrected *CCNE1* expression in CD8+ T cells (*P* = 3.5 × 10^−9^, *q* = 0.020), and the mPC-corrected *Ruminococcaceae UCG-003* abundance was negatively associated with gPC-corrected *SCAMP1* expression in CD19+ cells (*P* = 3.9 × 10^−9^, *q* = 0.020). *CCNE1* encodes cyclin E1, a positive cell cycle regulator (30, 31), while *SCAMP1* is involved in immune cell exocytosis (32) and endocytosis (33). Both *Lachnospiraceae* and *Ruminococcaceae* families have been frequently implicated in studies exploring the connection between the microbiome and systemic immunity, though the specific immune cells associated with these taxa varied between studies (34–36). Interestingly, identified associations were not significant prior to the correction for the main mPCs and gPCs (raw *P*-values > 10^−5^ before correction). This highlights that adjusting for principal components can help uncover hidden dependencies between microbiome composition and host gene expression.

## DISCUSSION

The relationship between the human intestinal microbial community and the immune system is one of the most intriguing and complex examples of host-microbiome interactions. The immune system normally maintains a balance between tolerance to commensal taxa and defense against pathogens. The mechanisms underlying how the microbiome influences the host’s intestinal immune system, as well as its role in inflammatory diseases, continue to be extensively studied (37). With an *in vitro* approach as one of the methodological solutions, it was shown that exposure of human colonocytes to gut microbiome resulted in widespread changes in the expression of genes involved in complex traits; as a proof-of-principle, a single microbe (*Collinsella*) was used to induce precise expression changes (38). In non-mammalian vertebrates (stickleback), observations of gut microbiome and host immune organ expression strongly suggest systemic impact of microbiome on immunity (39). Meanwhile, there remains a clear gap in robust analyses examining the “host-microbiome” interface in healthy human populations. In this study, we investigated these interactions by analyzing gut microbiome composition and host gene expression across intestinal tissues and immune cells within a large healthy cohort. Importantly, this cohort was particularly unique due to the exclusion of subjects with colon cancer, autoimmune conditions, or inflammatory diseases, providing a unique baseline for understanding microbiome-immune interactions in healthy individuals.

Linking the host transcriptome to the microbiome is inherently challenging due to the high dimensionality of the data. The sheer number of variables makes it difficult to distinguish true effects from random noise, often leading to few significant findings after multiple comparison corrections. In multi-omics studies, researchers may adopt a more relaxed significance threshold (e.g., *q* < 0.25–0.3) to capture suggestive associations. However, another effective strategy is to reduce the dimensionality of the analysis space, minimizing the number of statistical tests—for example, using PCA, cluster analysis, feature selection, and feature engineering (40–42). In this work, we employed an approach that incrementally increased the coarseness of microbial and host gene expression features. By aggregating features at higher levels, we reduced the number of tests and were able to detect broad, systemic effects, while lower-level aggregations allowed us to uncover more specific relationships between bacterial taxa and host gene expression. Our results revealed interesting associations at each level of the analysis, spanning both intestinal epithelial cells and blood immune cells. Some of these links obviously reflect the same interaction detected at different aggregation levels, while others may represent more level-specific phenomena.

One of the more prominent findings reproducible across different aggregation levels was the association between *Catenibacterium* and gene expression in immune cells, specifically CD8+ cells (Fig. 4B). The *Catenibacterium* genus belongs to the *Erysipelotrichaceae* family—an underexplored gut microbial family that has been linked to various aspects of host health. Considered rather immunogenic and related to host lipid metabolism (43), its members have been found to be overrepresented in the gut microbiome of HIV patients compared to healthy controls (44, 45). In these patients, *Erysipelotrichaceae* levels were positively associated with IL-1β levels and negatively associated with a marker of microbial translocation (levels of IgM against lipopolysaccharide core antigen) (44). Furthermore, a study on germ-free mice showed that colonization with *Erysipelotrichaceae* increases the expression of genes responsible for antigen presentation and immune system education (46). The *Erysipelotrichaceae* family was also associated with increased expression of extracellular matrix remodeling pathways in the intestinal tissue of IBD patients (11). Previously, we found that the proportions of *Catenibacterium*, alongside other highly immunogenic taxa, were increased in the gut of the indigenous Yakut population of Northeastern Siberia compared to the metropolitan population of Russia (47).

Interestingly, *Catenibacterium* was not only linked to host gene expression at the systemic level but also locally by showing a negative association with *MRPS21* expression in the transverse colon. The *MRPS21* gene encodes mitochondrial ribosomal protein S21, which plays a role in mitochondrial translation, and hence, its expression typically reflects overall mitochondrial metabolism. Mitochondrial dysfunction has been implicated in the pathogenesis of IBD (29, 48). A proteomic study on the onset of Crohn’s disease showed that the majority of mitochondrial proteins are downregulated in CD patients compared to healthy controls (27). This dysfunction is thought to lead to the production of reactive oxygen species (ROS), increasing intestinal permeability and promoting endotoxemia (28, 29). Supporting this, an *ex vivo* study demonstrated that ROS initiates transcytosis of commensal microbes in human colon tissue, a process that can be inhibited by a mitochondria-targeted antioxidant (49). Intriguingly, *Catenibacterium* and mPC11 were the only microbiome features in our study to be associated with gene expression in both intestinal cells and blood immune cells, reinforcing the potential importance of this genus in host-microbiome interactions across systemic and local immune responses.

The lack of significant associations with enterotypes suggests that the top-level microbiome features are not informative of host gene expression. However, a large-scale microbiome feature significantly associated with transverse colon expression for multiple host genes was the first microbiome principal component. Having analyzed its loadings, we discovered that it represents a gradient from common gut commensals (such as *Ruminococcaceae* and *Christensenellaceae*) to those that are either less typical for the colon (such as *Anaeroglobus*—usually found in the upper intestinal tract) and/or linked to inflammation ([*Ruminococcus*] *gnavus* from the *Lachnospiraceae* family and *Lachnoclostridium*) (50–52). The top negative loadings also included the *Brochothrix* genus, which, although not commonly found in the human gut, has occasionally been detected in stool samples (53), possibly as a transient microbe from food products (54). Interestingly, most taxa with positive loadings on mPC1 have recently emerged as biomarkers of metabolic and cardiovascular health, with some proposed mechanisms. For instance, the [*Eubacterium*] *coprostanoligenes* group is recognized for converting intestinal cholesterol to coprostanol, thus exerting serum cholesterol-lowering effects (55). Other important taxa, including the *Christensenellaceae R7* group, *Ruminococcaceae UCG-005*, and *Ruminococcaceae NK4A214* group, were identified among seven butyrate-producing taxa that were inversely associated with insulin resistance in a large cross-sectional study involving 2,166 participants (56).

The list of genes positively associated with mPC1 included *HMOX1*, *CBX6* (*q* < 0.05), and, at the edge of significance, *CPI17* (also known as *PPP1R14A*) (*q* = 0.0585). Both *HMOX1* and *CPI17* are thought to play protective roles in intestinal inflammation. *HMOX1* was shown to reduce inflammation through the induction of anti-inflammatory cytokine production (57). Experiments on germ-free animals showed that *HMOX1* expression is at least partially regulated by the gut microbiome (58). A by-product of *HMOX1*-encoded protein activity, carbon monoxide, was shown to ameliorate IBD symptoms, likely by reducing TNF-α expression (57, 59, 60). As for the *CPI17* (C-kinase potentiated protein phosphatase-1 inhibitor of 17 kDa), its expression is regulated by TNF-α (61). During intestinal inflammation, increased TNF-α levels result in reduced *CPI17* expression in intestinal smooth muscle (61). *CPI17* enhances smooth muscle contraction by inhibiting smooth muscle myosin phosphatase. A decrease in *CPI17* levels leads to decreased intestinal motility associated with IBD (62, 63).

On the other hand, *HSD3B2* and *SLC5A1* were among the genes negatively associated with the first microbiome principal component in the transverse colon. *HSD3B2* encodes the enzyme 3-Beta-HSD, typically found in hormone-producing tissues. However, recent studies suggest that mucus cells can also produce this enzyme and other steroidogenic enzymes, with their expression upregulated by TNF-α (64, 65). Our findings indicate that many of the genes associated with mPC1 are linked to TNF-α in various ways (Fig. 4A). Moreover, one of the taxa with negative loadings on mPC1, [*Ruminococcus*] *gnavus*, is known to produce a pro-inflammatory polysaccharide that induces TNF-α production by dendritic cells (66). This leads us to speculate that some of the mPC1-gene associations are mediated through cytokines, particularly TNF-α.

*SLC5A1* (*SGLT1*), another gene negatively associated with mPC1, encodes a transmembrane transporter responsible for dietary glucose and galactose absorption. Its expression is known to fluctuate with changes in lumen carbohydrate levels and is influenced by metabolic diseases (67) and inflammation (68). However, the direction of *SLC5A1* expression change in response to inflammation remains ambiguous (67, 69–71). Interestingly, in pigs, cecal microbiome composition was shown to explain 24% of the variation in serum glucose levels (72). Beyond their primary function in sugar uptake, glucose transporters are also involved in inflammation, malignancy, and the regulation of the gut microbiota (73).

Notably, the significant associations between microbiome features and gene expression were restricted to adaptive immune cells (T cells: CD8+ and CD4+; B cells: CD19+), while no associations were found with the examined components of the innate immune system (monocytes: CD14+; granulocytes: CD15+). For intestinal tissues, associations were observed only for the transverse colon host gene expression.

As a methodological limitation, we acknowledge that residualization may not fully account for correlations between predictors and covariates (age, sex, BMI, smoking status, collection date, intestinal site, and amplicon) and may slightly affect standard error estimates (74), though mathematically justified under certain conditions. However, because both microbiome and transcriptome descriptors were adjusted using the same strategy, the resulting associations represent covariate-independent relationships between the two data modalities, consistent with our goal of identifying intrinsic host-microbiome links. At the same time, residualization-based approaches are commonly used in microbiome association studies.

Our study demonstrates how a gradual dimensionality reduction approach can reveal novel associations between gut microbiome composition and host gene expression data. Most of the identified links, both in intestinal tissues and immune cells, were related to immunity, with some associations reproducible at multiple levels of hierarchical feature aggregation.

The identified associations provide promising candidates for experimental validation through methods such as culturomics, intestinal tract simulators with human colonocyte layers, or animal models. Establishing the specific effects of key microorganisms on host gene expression could lay the groundwork for precise microbiome-driven immune modulation using techniques such as fecal microbiota transplantation, live biotherapeutics, and other interventions. In the future, these insights may prove valuable for modulating systemic immunity.

## MATERIALS AND METHODS

### Study design and cohort enrollment

The study population consisted of 315 healthy individuals who visited the Academic Hospital of the University of Liège as part of a national colon cancer screening campaign. All participants were free of cancer, autoimmune diseases, or inflammatory diseases and were not taking corticosteroids or non-steroidal anti-inflammatory drugs, except for low-dose aspirin for thrombosis prevention (as described in reference 25).

### Sample collection and preparation

Biopsy samples for microbiome and host gene expression analysis were collected from three regions of the intestine: the ileum, transverse colon, and rectum. Immune cells were isolated from peripheral blood, following the protocol described in reference 25, and included six immune cell types from both the adaptive immune system (CD4+ T lymphocytes, CD8+ T lymphocytes, and CD19+ B lymphocytes) and the innate immune system (CD14+ monocytes and CD15+ granulocytes), as well as platelets.

### Normalization of microbiome data

The microbiome sequencing data used in this study have been previously published (13); details on DNA extraction, sequencing, and raw data processing are also available in that publication. A summary of the data processing workflow is provided in Fig. S7B.

Similarly to the data processing in the abovementioned article, the original taxon abundances produced by the taxonomy classifier were further subjected to removal of the determined contaminants, downsampling, and centered log ratio transformation (13). However, the subsequent mixed model data correction was performed in a different way, as described in detail below.

The data were subjected to a mixed model approximation implemented in the lme4 R package (75) with the following covariates and variables, and the residuals were collected to represent the corrected abundance of genera:


(1) $$\begin{array}{rl} genus\_abundance∼\; & age+sex+BMI+smoking\_status+\\ & (1|collection\_date)+intestinal\_site+Amplicon \end{array}$$

In the primary data analysis round, our aim was to determine the effects of the intestinal site and the 16S rRNA gene amplicon type on the observed taxon abundance measurements.

For the purpose of the amplicon (16S rRNA region) effect assessment, the abundances were corrected for all the variables of the model (equation 1) except for sample collection date, amplicon, and intestinal site. Then, the abundances of each taxon were compared across amplicon pairs (V1V2 to V3V4, V1V2 to V5V6, and V3V4 to V5V6) using Spearman correlation analysis. Further comparison of the amplicon pairs was performed using the Mann-Whitney *U* test.

In the process of determining the intestinal site effect on microbiome composition, the abundances were corrected for all the variables of the mixed model (equation 1) except for sample collection date and intestinal site. The homogeneity of variance was evaluated using the PERMDISP2 test (implemented via the betadisper function in the vegan R package (76). To assess general differences between intestinal sites, a PERMANOVA test (via the adonis2 function in vegan) was conducted.

Since the analysis showed no significant association between sample intestinal site and microbiome composition and most taxa exhibited high correlation between amplicons, for further analysis, we updated the mixed model correction method by adding intestinal site and amplicon to the list of variables we correct for. Thereafter, we averaged the adjusted abundances for each genus-subject pair across different amplicons and sites. To avoid overfitting, for genera with <150 non-zero values (prior to clr transformation) across all “sample-intestinal site-amplicon” combinations, the correction for intestinal site and amplicon was substituted by subtracting the mean abundance across them.

After these steps, one abundance profile per subject was obtained.

### Dimensionality reduction of microbiome data

Multiple strategies for dimensionality reduction were employed to derive microbiome features at various aggregation levels. After preprocessing and filtering, 145 genera were retained for analysis. The dimensionality reduction techniques applied included enterotyping, PCA, and clustering of co-abundant features.

Enterotyping was performed on taxonomic features after the removal of contaminants, without any transformations or corrections, using the DirichletMultinomial R package (77), with the optimal number of enterotypes (*n* = 4) selected using the Laplace goodness-of-fit criterion. Prior to enterotyping, the total read counts per sample were rarefied to 10,000 (for the V34 amplicon, data were rarefied to 5,000 reads, and then the counts were multiplied by two due to lower coverage data available for the region V34) and averaged across intestinal sites and amplicons. Outliers with very high median Jensen–Shannon divergence from 50% of closest neighbors (*P* < 0.01) were removed.

The PCA method was applied to corrected genus abundances using the ade4 R package (78). We selected the first 15 principal components of microbiome taxonomic abundances, which together explained 44.1% of the variance (further referred to as mPCs; Fig. S8).

To identify clusters of highly correlated genera, we constructed a correlation network based on corrected genus abundances with Spearman’s *⍴* ≥0.6. The Louvain algorithm, implemented in the igraph R package (79), was used to detect clusters within this network. The abundance of each cluster was calculated as the sum of abundances of its member taxa. Individual genera that did not belong to any of the identified clusters were retained as singletons.

To correct for the major signals in the microbiome data, we adjusted genus abundances using a linear model based on the first 15 mPCs and calculated the residuals for further analysis. These residuals, referred to as mPC-corrected abundances, were used in subsequent association analyses.

Overall, the following features were used to analyze microbiome associations with host gene expression:

1. Enterotypes (*N* = 4);
2. Microbial principal components (*N* = 15);
3. Microbial clusters and singleton genera (*N* = 7 clusters and 118 singletons);
4. mPC-corrected genera (*N* = 145).

### Host gene expression analysis

Human transcriptome data had been generated for the biopsy samples taken from the ileum, transverse colon, and rectum, as well as from the following immune cell types: CD4+ T lymphocytes, CD8+ T lymphocytes, CD19+ B lymphocytes, CD14+ monocytes, CD15+ granulocytes, and platelets using Illumina HT12 arrays as described in a previous publication (25). The data had been preprocessed, filtered, and corrected for covariates (sex, age, smoking status, and Sentrix ID). A summary of the data processing steps is shown in Fig. S7A.

Similar to the microbiome features, we employed several strategies for dimensionality reduction of the host transcriptome data. First, we utilized the classical principal components of gene expression data (referred to as gPCs), which were obtained from the same study (25). The number of gPCs varied slightly between different immune cell types, as the original paper selected PCs that maximized the number of *cis*-eQTLs (*N* = 27–59). Second, we performed a weighted gene co-expression network analysis (WGCNA) on the gene expression data corrected for covariates (from reference 25); parameters: minModuleSize = 30, soft thresholding power was evaluated by plotting scale-free topology fit index for each tissue/cell type and resulted in a range from 6 to 9. WGCNA identifies modules (clusters) of highly correlated genes, summarizing their expression values into module eigengenes (the first principal component of a given module) (80). Additionally, highly variable unclustered genes (those in the top quartile of expression variance distribution) were included as singletons. Finally, we obtained gene expression values corrected for the main principal components from reference 25.

In summary, the following transcriptomic features were used to analyze associations with microbiome features:

1. Host gene expression principal components (*N* = 27–59 gPCs depending on cell type and intestinal site, see reference 25);
2. Gene clusters and singleton genes (*N* = 14–47 clusters and 1,703–6,719 singletons depending on cell type and intestinal site);
3. Gene expression values corrected for the first 27–59 gPCs (gPC-corrected gene expression) (*N* = 6,638–15,401 depending on cell type and intestinal site).

### Analysis of associations between microbiome features and host gene expression

We explored potential associations between the microbiome and host transcriptomic features across different aggregation levels. The following relationships were modeled (Fig. 2):

1. gPCs, gene clusters + singleton genes, and gPC-corrected gene expression—with mPCs;
2. gPCs and gene clusters + singleton genes—with microbial clusters + singleton genera;
3. gPCs and gPC-corrected gene expression—with mPC-corrected genus abundances;
4. gPC-corrected gene expression and gene clusters + singleton genes—with enterotypes.

To detect associations, we used a linear regression model for each of the mentioned contrasts, except for the comparison between host genes and enterotypes: microbial feature ~ host gene expression feature.

Both microbial features and host transcriptomic features had been independently corrected for biological and technical covariates as described above. Before regression analysis, outliers were removed. Values were considered outliers if they fell outside the range of [Q1 − 5 × IQR; Q3 + 5 × IQR] for the feature distribution. The proportion of removed outliers was low (0.6% ± 1.4% per comparison; see Table S5), and in most comparisons (Table S6), no samples were removed. Multiple testing correction was applied for each type of comparison (e.g., gPC ~ mPC) separately for intestinal and immune cells using the Benjamini-Hochberg method. Associations with *q* < 0.05 were considered significant. To address the potential presence of non-independent comparisons being subject to multiple comparison adjustment, for each of our nine comparison blocks, we implemented an advanced procedure based on Sidak’s equation (81) that performs multiple comparison adjustment taking into account the effective number (Neff) of independent comparisons that is estimated from a *P*-value distribution derived from a large number of random permutations of the compared data (*k* = 1,000); after the Neff is calculated from Sidak slope, it is used for Sidak adjustment of the raw *P*-values. We also applied a global multiple testing procedure across all types of comparisons and all intestinal sites or immune cell types to highlight the most significant associations (Table S2).

Associations between enterotypes and gene expression data were assessed using the Kruskal–Wallis test. For comparison involving high dimensionality, the *q* values were additionally corrected for inflation using the genomic inflation factor (82). Significant associations were further validated using robust MM-type regression (*lmrob* function, method = “MM”) to mitigate the influence of residual outliers.

### Pathway enrichment analysis

To functionally characterize gene clusters of interest, we conducted over-representation analysis (ORA) using the Reactome Pathway Knowledgebase (83) via its embedded ORA online tool. For the gPCs of interest, we performed ORA on the 200 genes with the highest and the 200 genes with the lowest Spearman correlation coefficients per gPC. To mitigate potential bias caused by the overrepresentation of certain pathways in specific immune cell types (which could affect all principal components and result in the identification of cell type rather than cluster-/PC-specific pathways), we implemented a permutational control for all identified hits. Specifically, all transcripts were divided into bins of 100 elements each, based on their expression levels within the immune cell type of interest. We subsequently generated gene sets where each of the genes of interest was replaced with a random gene from the same bin. ORA was then applied to these permuted gene sets, and we retained as truly overrepresented only those pathways that were not overrepresented in the permuted gene sets (*N* = 1,000).

## Data Availability

The host gene expression data were previously published (47) and are available in the Array Express database (https://www.ebi.ac.uk/arrayexpress/) under accession number E-MTAB-6667. The microbiome data for 194 subjects were previously published (59) and are available in the European Nucleotide Archive (ENA) under BioProject accession PRJNA814419. The newly presented microbiome data for 121 subjects are available under the accession PRJEB111129. The essential data analysis code is available at https://github.com/Lev-genetik/CEDAR_mt.

## SUPPLEMENTAL MATERIAL

Supplemental figures spectrum.03593-25-s0001.docx Fig. S1 to S8.

Supplemental tables spectrum.03593-25-s0002.xlsx Tables S1 to S6.

ASM does not own the copyrights to Supplemental Material that may be linked to, or accessed through, an article. The authors have granted ASM a non-exclusive, world-wide license to publish the Supplemental Material files. Please contact the corresponding author directly for reuse.
