# Supplementary material for: Multi-level aggregation analysis of microbiome composition and host gene expression reveals associations with systemic and local immunity
Source: Microbiol Spectr. 2026 Aug 10;14(9):e03593-25. doi: 10.1128/spectrum.03593-25 (PMC13532277; doi:10.1128/spectrum.03593-25)
Supplement: Supplementary file 1 [file spectrum.03593-25-s0001.docx]

Multi-level aggregation analysis of microbiome composition and host gene expression reveals associations with systemic and local immunity

# Supplementary figures

**Figure S1 - Analysing impact of amplicon on variability of microbiome features.** A - human DNA fraction depending on amplicon; B - Contamination fraction depending on amplicon (see Methods); C - Cross-amplicon consistency dependence on taxa abundance (example for V12 and V34 amplicons); D - Spearman correlation between the genera abundances estimated using different amplicons.

**Figure S2 - Analysing impact of location on variability of microbiome features.** A - PCA for taxonomic composition, B - PCA for pathway abundances.

**Figure S3 - Top 10 genera of each enterotype sorted by average abundance.**

**Figure S4 - Top 10 expressed genes in different intestinal locations and blood cells.** Y axis reflects log2 transformed and robust spline normalised fluorescence intensities (Momozawa et al. 2018) after correction for covariates.

**Figure S5 - Associations between microbiome composition and host gene expression: the genera involved in only 1 association.** Extension of the results presented in Figure 3.

**Figure S6 - Scatterplots illustration top non-significant associations between 1st taxonomic principal component and gene expression with 0.05<FDR<0.15.** TR - transverse colon, mPC - microbiome principal component.

**Figure S7 - Workflows of data preprocessing prior to analysis of associations.** A) Gene expression analysis. B) Taxonomic analysis of the microbiome.

**Figure S8 - Microbiome data: variance explained by the leading principal components (mPCs).** A) Per-component variance for 20 first components. B) Cumulative variance, with the dashed lines indicating the 15^th^ component.

# Supplementary tables

**Table S1.** List of potential contaminants.

**Table S2.** Associations between taxonomic composition and gene expression on different levels of data aggregation.

**Table S3.** Top 200 genes positively correlated and top 200 genes negatively correlated with CD8+ gPC5.

**Table S4.** Modules of highly correlated genes detected with WGCNA which were included in significant and suggestive associations (see Supplementary table 3).

**Table S5.** Number of the samples removed as outliers during each comparison.

**Table S6.** Number of nominal tests performed for each of 9 comparison blocks.
